# A New Primary in Parotid Gland with History of Treated Mediastinal Solitary Fibrous Tumour

**DOI:** 10.1155/2019/3234692

**Published:** 2019-01-06

**Authors:** Siddiq Ahmed, Syed Iftikhar Ali, Faizan Farid, Muhammad Ali, Waqas Ahmed, Talat Youseef

**Affiliations:** ^1^Oral and Maxillofacial Surgery Department, Leicester Royal Infirmary, University Hospitals of Leicester NHS Trust, Infirmary Square, Leicester LE1 5WW, UK; ^2^Plastic Surgery Department, Combined Military Hospital, Rawalpindi, Pakistan; ^3^Oral and Maxillofacial Surgery Department, Indus Hospital, Muzaffargarh, Pakistan; ^4^Radiology Department, St George's University Hospitals NHS Foundation Trust, London SW17 0QT, UK

## Abstract

Solitary fibrous tumours (SFTs) are rare tumours in the head and neck region. They have been reported in many anatomic sites but occurrence in the parotid gland is exceptional. We report a very rare finding of a benign SFT of the parotid gland in a patient with a past history of excision of a malignant type of mediastinal tumour. It is important that clinicians are aware of the possible existence of SFT in the parotid as a synchronous lesion or occurrence of the same disease later on elsewhere when SFT is diagnosed at one anatomical site. This case report illustrates that regular clinical and imaging follow-up is essential in SFTs to look for the appearance of new lesions in the other anatomic site.

## 1. Introduction

Solitary fibrous tumour (SFT), also termed as “haemangiopericytoma,” is a neoplasm of mesenchymal origin likely from adult mesenchymal stem cells [1].

SFTs are classified as benign or “typical” and “malignant” based on histopathological features [2]. They are usually benign and are treated by surgical resection. Late recurrences at local or at distant site are one of the clinical characteristics of SFTs [3]. The anatomical origin of SFT is variable. SFT was first described by Klemperer and Rabin in 1931 as pleural mesothelioma [4], and since then it has consistently been found mostly in the pleura and also in other anatomical locations including the head and neck region [5].

SFT in the parotid gland is uncommon and very few cases of parotid SFT are reported. Degnan et al. reported malignant abdominal SFTs in a patient who had complete resection of a benign intracranial SFT in the past [6]. To the best of our knowledge, there is no previous report of a benign or malignant parotid SFT in a patient with a history of any type of previous SFT diagnosed or treated in any other anatomic location. Due to the unavailability of any previous such finding, the possibility of the presence of SFTs in the parotid can be overlooked when the intra- or extrathoracic SFTs are investigated and treated. Early identification and treatment of these tumours may reduce the extent of surgical resection and subsequent related complications. We report a rare case of SFT arising in the superficial part of the parotid gland with a history of excision of a malignant type of mediastinal tumour more than a decade ago.

## 2. Case Report

**Figure 1:**
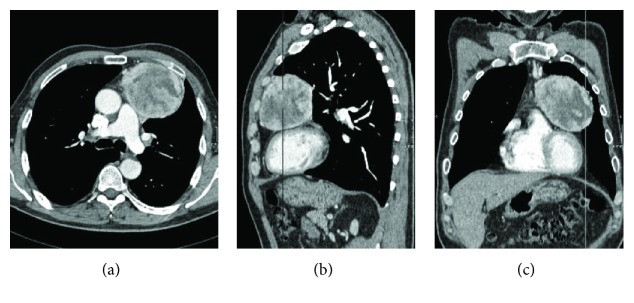
Contrast-enhanced CT of the chest. (a) Axial, (b) sagittal, and (c) coronal reformatted images revealing a well-defined anterior mediastinal mass, abutting the heart showing heterogeneous enhancement with pericardial invasion without any evidence of myocardial, aortic, or pulmonary artery involvement.

**Figure 2:**
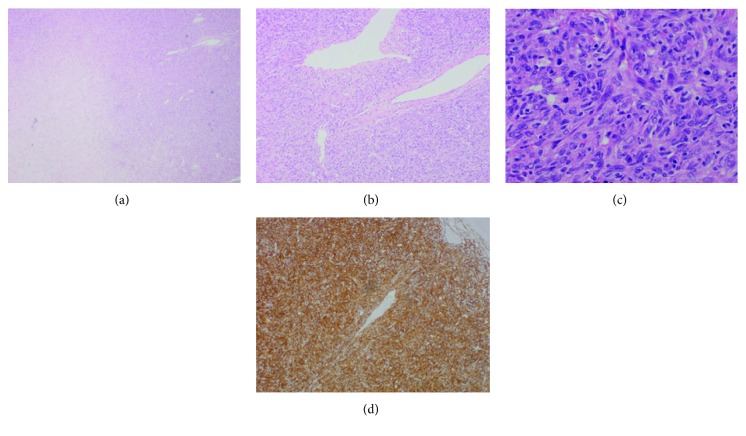
(a) Microscopic examination of the excised mediastinal lesion demonstrating tumour necrosis. (b) Spindle cells with haemangiopericytomatous pattern. (c) Moderate cytological atypia and mitoses. (d) Strong positive immunohistochemical staining for CD34.

A 79-year-old man presented with gradually enlarging painless swelling in the left parotid region over an 8-month duration. Past medical history revealed that he was treated 11 years ago for a malignant SFT in the anterior mediastinum (Figures 1 and 2) by complete excision followed by radiotherapy. He was regularly followed up every year for mediastinal disease with clinical and radiological examination. Since there was no clinical or radiological evidence of new disease or recurrence on follow-up for 10 years, he was later discharged from the care.

**Figure 3:**
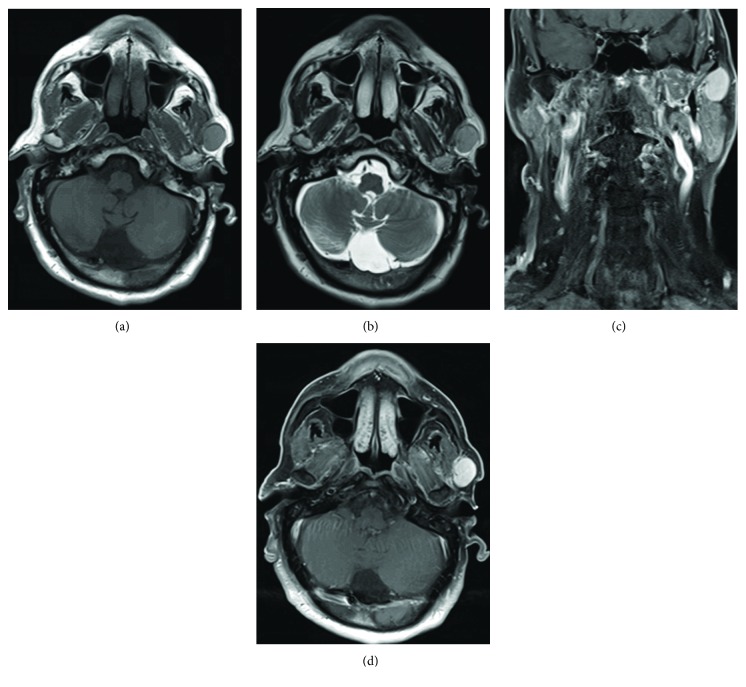
(a–d) MRI neck. (a, b) The mass at the left parotid space with slightly high signal intensity to the muscles on both axial T1 and T2 spin echo MR weighted images. (c, d) Coronal and axial postcontrast axial fat suppressed T1 MR weighted images demonstrate avid homogenous enhancement of the tumour.

**Figure 4:**
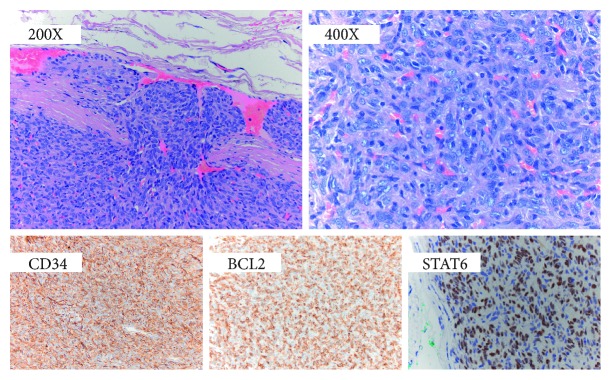
Hematoxylin and eosin staining at low magnification (200x) and high magnification (400x) showing a solid tumour in the parotid gland, with cords and nests of spindle-type cells. Immunoperoxidase staining positive for CD34 and BCL-2. STAT6 immunostain shows diffuse staining of the SFT cells.

On clinical examination of this new left parotid lump, a 3 × 3 cm mass in the left parotid with no overlying inflammation was found. The lesion was well circumscribed, not tender, and soft in consistency. There was no palpable cervical lymphadenopathy. The rest of the clinical examination was unremarkable. Ultrasound imaging revealed well-defined pseudocystic lesion within the superficial lobe of the left parotid gland. Magnetic Resonance Imaging (MRI) also demonstrated a well-defined mass within the left parotid arising likely from the parotid fascia with no evidence of parenchymal or neurovascular invasion. The lesion showed high signal intensity on T1- and T2-weighted images and homogeneous enhancement postcontrast and restricted diffusion (Figure 3). The right parotid and submandibular glands appeared normal. No cervical lymphadenopathy was found. Fine-needle aspirate was nondiagnostic. Radiological examination of other potential SFT sites did not reveal any pathology. Histopathological examination of tumour (Figure 4) following left-sided superficial parotidectomy showed plump spindle-shaped cells with indistinct cytoplasmic borders and some variation in nuclear size. There was prominent admixed vascular component composed of thin-walled channels with infrequently and vaguely haemangiopericytomatous appearance. Tumour necrosis and high mitotic activity seen with malignant lesions were not observed. Immunohistochemistry indicated diffuse strong expression of CD34, BCL-2, and CD99 and showed nuclear expression with a punctuate morphology for STAT6. Histopathological findings were confirmatory of SFT.

## 3. Discussion

SFTs are slow-growing neoplasms that manifest as painless masses. They commonly occur in middle age, with no gender difference. SFTs are mostly benign with good prognosis. Their behaviour in terms of local or distant relapse, metastasis to extrathoracic sites, or malignant changes has been well described [7, 8]. This unusual nature of SFT in terms of its origin or new appearance at various other sites in the body has implications for clinical management.

Occurrence of SFT in the parotid gland is very rare [9–12]. This case represents even more unusual finding of SFT in the mediastinum and then later in the parotid gland. To the best of our knowledge, such pattern of occurrence of SFT as a new primary has not been previously reported in literature. This report highlights the importance of detailed history, meticulous clinical examination, and advanced imaging studies to rule out the possibility of existence of single or multiple synchronous lesions when SFT is diagnosed at any anatomic site.

Computed tomography (CT) and MRI are the two main imaging aids in the diagnosis of SFT [13]. Typical CT features include well-defined ovoid mass, isodense to the muscles, whereas on MRI it appears isointense to the muscles on T1-weighted images, mildly hyperintense on T2-weighted images and diffusion restriction on DW1. The tumour may show either homogenous or heterogeneous enhancement on both contrast-enhanced CT and MRI. On dynamic contrast-enhanced MRI and dual-phase CT, time-intensity curves (TICs) exhibit additional diagnostic features of rapid enhancement and slow washout pattern [14].

Surgery remains the mainstay of the management for this type of neoplasm whereas radio chemotherapy is the alternative treatment of choice when tumour resection is not the ideal option [15]. SFTs predominantly exhibit nonmalignant behaviour when they occur in the parotid gland region but can be aggressive and grow in size significantly [16, 17] in which case extensive surgery may be required with greater risk of complications afterwards. In our case, owing to its involvement with only the superficial part of the parotid, the SFT was completely excised with adequate tumour-free margins.

There is a possibility that in our case a concurrent SFT in the parotid gland existed at the time of management of the mediastinal SFT. It is difficult to prove this in retrospect due to the unavailability of imaging in the head and neck region or of other potential areas in the body where SFT could develop or recur when the diagnosis of mediastinal SFT was made. Early diagnosis of SFT may limit the extent of surgical or nonsurgical treatment. Stratification and risk assessment strategies pertinent to the behaviour of SFT have been introduced recently [18]. Comprehensive clinical management guidelines covering metastatic SFT are also available [19] but there is a lack of uniform policy addressing the benign type of SFTs.

Establishing a working diagnosis plays a central role in the management of SFTs. This can be achieved to a reasonable extent by MRI as it shows variable intensity signals on T1- and T2-weighted images. Although nonspecific, they are important radiological features of SFTs depending on the quantity of their collagen content [13]. Even though there are cost implications, routine screening by MRI of all sites with a potential of SFT development can be a valuable and safe strategy.

The incidence of late recurrence of SFT at extrapleural sites varies. Bauer et al. [9] reported that the median time to first recurrence of SFTs was 12 years (range: 10-23 years). In our case, the interval between mediastinal SFT and parotid tumour was also more than a decade. Although we have found SFT in the parotid as a new primary rather than recurrence but due to highly unpredictable behaviour of SFTs and possibility of their recurrence or relapse, long-term surveillance is also necessary for benign SFTs. We propose an annual combined clinical and radiologic follow-up for the rest of their lives for patients treated for SFT. Further research is needed to validate such suggestion and to set guidelines for diagnosis, treatment, and continued surveillance in patients diagnosed with SFT even with benign pathology.

## 4. Conclusions

Clinicians should be aware of the possible existence of parotid SFT in a patient with a history of SFT in any other anatomic location. Earlier diagnosis of SFT through clinical and advanced imaging examination of high-risk sites can minimize the extent of required treatment.
